# The Effect of Law Students in Entrepreneurial Psychology Under the Artificial Intelligence Technology

**DOI:** 10.3389/fpsyg.2021.731713

**Published:** 2021-11-03

**Authors:** Chengjin Xu, Zhe Zhang

**Affiliations:** ^1^School of Law, Shandong Normal University, Jinan, China; ^2^School of Management Science and Engineering, Shandong University of Finance and Economics, Jinan, China

**Keywords:** artificial intelligence, neural network, college students' entrepreneurial psychology, wavelet transform, psychological capital

## Abstract

With the increasingly serious employment situation in China, the government and schools encourage college students to start businesses to alleviate employment pressure. College student's successful entrepreneurship depends on national preferential policies, social support, and, most importantly, their healthy and solid psychological quality and entrepreneurial psychological quality. The purpose is to understand the entrepreneurial psychology of college students and study the entrepreneurial psychological effect. Firstly, the four aspects of entrepreneurial psychology are summarized, including entrepreneurial awareness, entrepreneurial volition, entrepreneurial ability, and entrepreneurial personality. Secondly, the research status of college students' entrepreneurial psychology is reviewed, and the existing problems are pointed out. Thirdly, the combined model of wavelet transform and Neural Network (NN) is proposed, and the feasibility of the proposed model is evaluated through the analysis of college students' entrepreneurial psychology. The wavelet NN is used in experimental design to predict college students' entrepreneurial psychology, and the predicted results are compared with the actual value. From the perspective of the prediction results of entrepreneurial psychology, the combination of wavelet algorithm and neural network is more accurate for entrepreneurial psychology prediction and evaluation results of law students. Overall, the difference between the predicted value and the actual value is within 0.3 points, which is relatively stable. According to the analysis of single-factor results, the scores of students of different majors in the four dimensions of entrepreneurial psychology are all higher than 3.5, but there is no significant difference among the four dimensions (*P* > 0.05), indicating that the major has no significant impact on entrepreneurial psychology; law students with different educational backgrounds have significant differences in entrepreneurial psychology (*P* < 0.05), among which students with a master's degree have the strongest entrepreneurial will, while doctoral students have the lowest entrepreneurial will; in terms of entrepreneurial psychological capital, men's self-efficacy is higher than women's, and the difference is significant (*P* < 0.05). The difference between males and females in the scores of entrepreneurial psychological factors' four aspects is not very obvious. In terms of entrepreneurial psychological capital, males' self-efficacy is significantly higher than females' (*P* < 0.05). Artificial Intelligence (AI) technology has great application prospects in the prediction and evaluation of college students' entrepreneurial psychology, and college students' entrepreneurial psychology is highly correlated with gender and education.

## Introduction

With the continuous acceleration of China's modernization drive, people's material needs and living standards have been greatly satisfied and improved, while social pressure is accumulating on the young people. College students, as the largest younger population, are under pressure from all aspects even though they haven't entered society. In particular, college students' psychological pressure is growing. Presently, Chinese college students' psychological situation is mainly evaluated through various evaluation scales, such as Questionnaire Survey (QS), psychological survey report, and social survey report (Chen and Yu, 2020). Meanwhile, the survey perspectives are from psychology, sociology, and university administrators, subjective interpretation and objective analysis are conducted on the psychological state of college students, and students' psychological factors and potential risks are studied (Fan et al., 2021). However, these survey methods are independent in form and content, the results are not compatible and not integral, and the content is not comprehensive. Besides, there are also deficiencies in reliability and validity. The conventional psychological survey report lacks timeliness. With the advent of the big data era and the popularity of the Internet, data acquisition and processing have become more convenient and efficient. Many domestic researchers begin to apply Artificial Intelligence (AI), Deep Learning (DL), and Neural Network (NN) technologies to psychological evaluations, thereby developing a new method for college students' mental health evaluation. Wu and Song (2019) improved students' participation with CRS technology in acquiring entrepreneurial knowledge, which was an effective tool to improve students' entrepreneurial ability. Wu and Yuan (2018) helped students cultivate entrepreneurship and enhance the effectiveness of conventional education with web-based (Institute of Computing Technology, ICT) tools. Obviously, the application of big data and AI has gradually matured, and some achievements have been made in the education and ability training of college students. With the employment problem of college students getting serious, the government, society, and higher education institutions are actively promoting college students' entrepreneurship encouraging college students to achieve self-gratification using the development trend of the times and economy (Nikpanah et al., 2021). These measures can solve the employment problem and promote economic growth. As of last year, 70% of college students have an entrepreneurial intention, but with less than a 10% success rate. Thus, the government and schools have to investigate the entrepreneurial psychology of college students (Li et al., 2020). Relevant surveys suggest the main reason for college students' entrepreneurial failure is the lack of psychological construction: pessimistic emotions hinder calm judgment in the entrepreneurship process (Chen et al., 2021). Although entrepreneurship can alleviate college students' employment pressure, the lack of positive and healthy entrepreneurial psychology contributes greatly to the continued depression of the success rate of college students' entrepreneurship in recent years. College students' entrepreneurial difficulty can be solved only if students' entrepreneurial enthusiasm and entrepreneurial psychology are enhanced, and a correct entrepreneurial concept is established (Guo et al., 2021).

In the developed world of Europe and America, entrepreneurship education has seen relatively early development, and relevant research has been matured. Moreover, while undertaking entrepreneurship education, these countries have gradually realized that college students' entrepreneurship can effectively promote socio-economic development (Shaheen et al., 2020). Therefore, international research on entrepreneurship cognition, entrepreneurship education, and entrepreneurship psychological quality are reviewed to better understand the development process of entrepreneurship education, which is of great significance to the unique research direction of this paper, but also of great reference values to the development of entrepreneurship education in China (Yuan et al., 2020). Daniella et al. (2021) believed that the personality traits and entrepreneurial results of entrepreneurs ultimately determine the essence of entrepreneurship. The entrepreneur's character and entrepreneurial results determine the essence of entrepreneurship. Li et al. (2021) believed that “entrepreneurship education is a process to provide students with the ability to recognize business opportunities and enable them to have the insight, ego, knowledge, and skills required for entrepreneurial actions.” Entrepreneurship education was the integration of various forms and multi-stage organizations and enterprises, as well as an important channel to help entrepreneurs achieve their life goals and realize their life values. In today's society, the research on entrepreneurship education is deepening and developing.

To explore the entrepreneurial psychological effect on college students, help college students better understand entrepreneurship, and improve the success rate of entrepreneurship, the psychological factors of college students' entrepreneurship are evaluated and predicted based on AI technology. Here, innovatively, the wavelet transform is combined with the neural network algorithm to establish the analysis and prediction model of college students' entrepreneurial psychology under the background of DL; College students' psychological data and personal information are used as samples to perform a more scientific and reasonable prediction of their entrepreneurial psychology and verify the accuracy of wavelet neural network by comparing the results with the actual situation. The results are used for analysis and evaluation of college students' entrepreneurial psychology, and the entrepreneurial psychology is evaluated through the single factor chi-square test. The results can guide the current college students' entrepreneurship education and improve the success rate of entrepreneurship. The innovation is that combining the characteristics of college students, through the four sub-dimensions of entrepreneurial intention, entrepreneurial will, entrepreneurial ability, and entrepreneurial personality, and using first-hand data to explore the entrepreneurial psychological quality of college students majoring in law. The study meantime proves the influence of the environment on the psychological quality of entrepreneurship, especially the social-cultural environment, and has a large effect on the formation of the psychological quality of entrepreneurship of college students. Moreover, it draws more meaningful conclusions and enriches other scholars' research on the psychological quality of undergraduates' entrepreneurship, providing a reference for future research on the psychological quality of undergraduates' entrepreneurship.

## College Students' Entrepreneurial Psychology

### Overview of Entrepreneurial Psychology

Entrepreneurial psychology is a comprehensive long-term psychological quality, which is formed under different educational and growth environments and can support and guide an individual's entrepreneurial behaviors. Besides, a comprehensive and integral entrepreneurial psychology can help individuals complete entrepreneurial behaviors (Carvalho et al., 2021). Entrepreneurial psychology includes four aspects: entrepreneurial awareness, entrepreneurial volition, entrepreneurial ability, and entrepreneurial personality. Formally, entrepreneurial psychology is the psychological quality slowly exercised by college students in the process of growth and learning under the external environment, education, and self-efforts. Meanwhile, entrepreneurial psychology is reflected in the process of entrepreneurship through ability and awareness. Presently, to ensure successful college students' entrepreneurship, college students' psychological quality must be jointly promoted from many aspects, such as family, school, and society (Guo and Guo, 2020).

(1) Entrepreneurial awareness. Generally, entrepreneurial awareness is the entrepreneurial intention and entrepreneurial cognition of entrepreneurs. Entrepreneurial awareness is the individual's subjective tendency and psychological activities for entrepreneurial activities, including entrepreneurial motivation, entrepreneurial thinking, business thinking, and ideals and beliefs. Once these kinds of awareness are formed in students' minds, college students' entrepreneurial potential and success rate of entrepreneurship can be greatly improved (Mu et al., 2020).
(2) Entrepreneurial volition. Volition is one of the most important features distinguishing humans from animals. An individual's behaviors are often determined by their volitions, while their volitions guide them to achieve specific purposes. Similarly, entrepreneurial volition is the guidance for individuals to achieve entrepreneurial goals. Entrepreneurial volition is manifested in perseverance, self-progress, self-reliance, and toughness. Besides, entrepreneurial volition plays an important coordinating and integrating role in entrepreneurship. Firstly, entrepreneurial volition encourages behaviors that help entrepreneurship. Secondly, entrepreneurial volition resists and overcomes negative acts that harm entrepreneurship (Ting, 2020).
(3) Entrepreneurial ability. Entrepreneurial ability refers to the ability of entrepreneurs to develop or create a new research field or creative things by integrating their knowledge, experience, and skills, and entrepreneurial ability show sustained and stable psychological process in entrepreneurship. Entrepreneurial ability includes the following aspects: the ability to create new ideas and new thoughts, the ability to operate and manage, the ability to cooperate with others, the ability to determine things, and the ability to learn by oneself (Sabir et al., 2021).
(4) Entrepreneurial personality. Entrepreneurial personality is a stable psychological quality of entrepreneurs formed under education and social practice based on physiological quality. Hamburg psychologist Berg hart Andreas thinks adventurous spirit is the sixth most important factor affecting personality. People with an adventurous spirit will fear nothing in entrepreneurship. Even if they encounter difficulties, they will take the initiative to accept challenges with confidence and go forward bravely (Ilyas et al., 2021).

### Feasibility of Application of AI Technology to College Students' Psychological Evaluation

At present, the advantages of the AI technological application can be illustrated in three aspects: test assistance, classification analysis, and early warning research judgment. 1. AI technology can provide accurate and intelligent on-demand evaluation services through intelligent auxiliaries. 2. The application of AI technology can reduce repetitive works in psychological counseling, such as QS statistical analysis, thereby improving the speed and efficiency of the evaluation scale and QS analysis. 3. The data source of the existing AI auxiliary system is the ready-made student information, and its working principle is still based on the comparison and cross-analysis of the existing student data. Thus, the extracted information should be structured first. Obviously, most data sources are structured. Plenty of unstructured information is closer to students' mental state but requires data structures and data cleansing. The cleaned data can predict the corresponding evaluation results according to algorithm models.

The rapid development of technology has brought great convenience to people's work and life. In the process of deep integration with human survival and the environment, it also shows complexity and particularity different from the previous environment, which provides new research ideas and methods for psychological research. Psychology is a science that studies the laws of human behavior and mental activity. Since the emergence of human beings, there has been concern about human psychology and behavior (Sivakumar et al., 2021). Psychology analyzes human behavior and psychology through scientific methods, including observation methods, survey methods (questionnaire methods and interview methods), test methods, and experimental methods. These methods are easily affected by the expectations or motivations of the subject and the subjects themselves and produce false or confusing results, that is, internal validity may be affected. Because it is a limited and representative selection within a certain period, the external validity and effectiveness of these methods are also questioned. At present, psychological research mainly uses self-reporting or subjective observation as the main technical means to study people's behavior and psychology in real or online environments (Xu et al., 2021). With the development of technology, the network environment can no longer be simply regarded as a tool. It has been deeply integrated with human survival and its environment. It has a completely different complexity and particularity from the previous traditional psychology research environment, and it also provides new ideas. The advent of the era of artificial intelligence and big data has opened a new door for the research of psychology (Wu and Mao, 2020). With the popularization of the Internet and various smart wearable devices, the virtual environment and real-life continue to merge, and various psychological and behavioral phenomena of people in the real society can be electronically recorded as big data and stored, such as network access behaviors, social emotions, social attitudes, and mental health issues (Jiang and Zhi, 2021). Researchers use the data left by users to predict their personality traits or behaviors. Big data not only brings new topics to psychologists in the research content, but more importantly, the combination of big data and artificial intelligence can use ecological behavior data, and combined with artificial intelligence technology, it realizes the automatic identification of people's psychological indicators, that is, ecological recognition, which greatly expands the scope of psychological research and application.

The proposal of ecological recognition provides psychologists with new research tools and perspectives. It provides new possibilities for understanding personality, well-being, suicide intervention, and even analysis of social hot events in the Internet age. By digging into the behavior and psychology of individuals and groups in different environments, it can help achieve large-scale, real-time description, prediction, explanation, and control of people's mental health or social attitudes, thereby effectively preventing risks. With the continuous iterative update of computer software and hardware, the application of big data in psychology will surely be more in-depth in the future, such as the application of wearable devices and somatosensory devices. In addition to its irreplaceable potential in psychological experiments, artificial intelligence will also greatly expand the field of psychology in other aspects. With the continuous development of smart terminals and the continuous acceleration of mobile data, artificial intelligence can not only provide a platform for psychological intervention, but it is also more likely to become the main force of psychological intervention. Scientific research methods separate psychology from philosophy, while artificial intelligence and big data may bring psychology into life more deeply (Daniella et al., 2021).

Students' psychological evaluation is based on a comprehensive survey of college students' past behaviors and personality characteristics to predict their future behavioral tendencies (Yin et al., 2020). AI technology can analyze existing data, form models, and analyze and predict future data. The application of AI technology in college students' psychological evaluation should be based on the deep understanding of human groups, individual behavior, and psychological laws (Jiang et al., 2021). The instrumentality of technology is to help people become proficient in specific fields. Overall, AI technology can evaluate college students' psychological status in terms of the explicit aspect of technology or the implicit aspect of psychoanalysis philosophy (Wachter et al., 2021). The AI technology reduces information entropy and finds possible certainty from countless uncertainties. By comparison, AI technology analyzes and compares the historical data of groups and individuals based on a full data sampling analysis, and then predicts the psychological state of the respondents using intelligent methods, such as the DL algorithms (Xi et al., 2021).

(1) Many studies have proved that AI technology is more accurate, efficient, and comprehensive in grasping various psychological factors and their weights. In conventional psychological evaluation, the general idea of scale design and implementation is the verification of the proposed hypotheses. Hence, the selection of evaluation factors and the weight of factor weights are affected by subjective judgment and professional background. If external conditions, such as human, material, and financial resources are limited, the design of the scale and the effectiveness, reliability, and validity of the evaluation will be immeasurable. The difference between college students' individual psychological factors and group data is excavated through comparison and cross-analysis, and the threshold to measure the psychological state of college students is obtained. For college students, these thresholds can be viewed at will. Thus, when the warning threshold is exceeded, the decision-maker can take corresponding measures according to the warning level. Therefore, AI technology plays an active and powerful role in analyzing big data, identifying data correlations, and inferring possible causal relationships between data factors (Ouyang et al., 2021).
(2) The strong adaptability of AI technology can effectively solve the timeliness and personalization of psychological evaluation. Besides, real-time evaluation is critical for campus psychological crisis, so the timeliness of evaluation results should be very high. College students' psychological state fluctuates greatly and may not be fully matured (Huang Z. et al., 2021; Huang Y. et al., 2021). The ever-changing social environment may incentivize college students' psychological instability, so various factors of college students' psychological evaluation should be coordinated (Ren et al., 2021). As a saying goes that change itself is the only constant. Thus, AI is a dynamic process and is in line with the dynamic characteristics of college students' psychological evaluation. Comparatively, based on big data analysis, AI technology comprehensively sample the data of the respondents, intelligently correct and update influencing factors and weights, and dynamically correct and optimize the iterative test model using the DL training. Meanwhile, the reliability and effectiveness of the system are continuously evaluated. Simply speaking, AI technology can identify the fluctuation of relevant factors, correct the weight of factors, and update the algorithm in automatic correction, thereby responding to the changes of college students' psychological evaluation factors, reflecting the evaluation results, and recommending early warning.

### Wavelet NN

There are four steps for the application of AI technology. 1. Cause determination for the problem. 2. Original sample data acquisition, screening, and preprocessing. 3. Establishment of the algorithm model and design of the learning and training process. 4. The results output and solutions proposal. Of these steps, the acquisition of original sample data needs data cleaning, because the samples are often randomly selected unstructured data which should be transformed into structured data. In this process, correction and cleaning may be considered. At the same time, the output results need to be normalized to prevent data generalization and over-fitting. Finally, all data are uploaded to the cloud to form a database, and the AI is realized through the calculation and analysis of big data technology. Meanwhile, to protect students' privacy, data should be desensitized, and at the same time, system security protection measures should be strengthened to avoid data leakage (Huang and Li, 2021).

There are plenty of conventional methods to predict and evaluate college students' psychological state, with different evaluation indexes while without a unified and standardized evaluation result. AI technology is widely used, has high prediction accuracy, and can save manpower and material resources compared with the QS.

The most important problem in neural network prediction is the structure of the neural network. Generally, the input data and output data can be determined based on the sample data of the specific problem and the nature of the problem itself (Urooj et al., 2021). Use part of the data as training data to train the neural network, and the other part of the data to verify the performance of the network. In this way, parameter settings such as the number of hidden layers of the neural network applicable to the sample data, the number of nodes in each layer, and the connection weights of each layer are determined. Finally, use the remaining part of the data to input the trained neural network model to get the output, that is, the predicted value. The neural network recognizes the characteristics of complex non-linear systems and has a better effect on short-term traffic flow data prediction (Sergent et al., 2020). From the beginning, a single neural network model uses different neural network models for combined forecasting, to the combination of neural network models and other model methods for forecasting, complementing each other and improving the accuracy of prediction. However, the shortcomings of neural networks are also obvious (Wang et al., 2021). Due to the “black box” learning model, sufficient raw data are required to train the model. If the data are insufficient, it is very likely that there are no “learning really useful things, and then not getting what you want.” The local minima and slow convergence during the training process are the defects of the neural network method itself, and the fixation of parameters is often only suitable for current research samples. Based on the various problems and shortcomings of neural networks, many improved different neural networks have been developed later, which greatly improves the promotion and application of neural networks. The past confirmatory research has transitioned to the current practical stage. Although the scope of application and application conditions of the neural network is still explored, its broad development prospects can already be seen (Du et al., 2021).

In terms of NN algorithm selection, the BP algorithm has slow convergence speed, different network structure selection, and excessive dependence on experience, so the results may get over-fitted. Thus, to overcome the shortcomings of BPNN, the wavelet NN is selected as the main algorithm of the prediction model. Firstly, the wavelet NN element and the whole structure are determined based on the wavelet analysis theory, which can avoid the blindness of BPNN in structural design. Secondly, the wavelet NN has a stronger learning ability and higher accuracy. In general, given the same learning task, the wavelet has a simpler structure, faster convergence speed, and higher accuracy. Therefore, the wavelet NN is more suitable for the exploration of entrepreneurial psychology affected by human factors, and the prediction results can be dynamically updated in real-time (Zhang et al., 2020).

Wave-turned NN is an emerging mathematical modeling and analysis method in recent years, which is formed by the combination of traditional NN and wave-variable function. Wavelet analysis is also a new analytical method, but it is more mature than the development of chaos theory (Hgg, 2020). It has been successfully applied in differential equations, fractal recognition, approximation theory, computer vision, and non-linear science. Especially for non-stationary time series, the time-frequency nature of wavelets has been well-demonstrated in analysis and prediction.

Wavelet analysis is milestone progress in the development history of Fourier analysis. Wavelet analysis is considered to be the essence of work in the field of harmonic analysis for half a century. In recent years, it has become the focus of attention of many disciplines in many countries (Sukirno et al., 2020). Wavelet analysis has been widely used in speech recognition, information processing, image processing, celestial body recognition, and other fields. Wavelet analysis not only has good localization characteristics in both time domain and frequency domain analysis, but also uses gradually refined time domain and spatial domain sampling steps for the high-frequency components of the signal, so any details of the signal can be analyzed. Wavelet analysis overcomes some unknowns of Fourier analysis, and it makes up for the shortcomings of Fourier transform that can only analyze steady-state signals. Wavelet has great applicability as a tool for signal processing and signals analysis, and meanwhile, it has achieved more and more extensive and more in-depth applications in various fields (Zheng et al., 2021).

Wavelet analysis technology is an effective tool for time-domain and frequency-domain analysis of unsteady signals. Suppose the wavelet function is Ψ(*x*), which satisfies the following conditions in the constituent space, as (1):


(1) $$\begin{array}{ll} C_{\psi }=\int_{R^{*}}\frac{|\hat{\psi }(w){|}^{2}}{\left|w\right|}dw<\infty & \;(1) \end{array}$$

R^*^ represents all non-zero real numbers, $\hat{\psi }\left(w\right)$ is the Fourier transform of ψ(*x*), and Ψ(*x*) indicates the wavelet mother function. The wavelet mother function is shown as (2):


(2) $$\begin{array}{l} \psi \left(a,b\right)\left(x\right)=\frac{1}{\sqrt{\left|a\right|}}\psi \left(\frac{x-b}{a}\right) \end{array}$$

The continuous wavelet function is generated by the wavelet mother function ψ(*x*) after transforming the parameter (a,b). Among them: *a* represents the expansion and contraction factor; *b* represents the translation factor.

Meanwhile, wavelet NN has self-learning ability, can maintain local temporal changes, and has the properties and classification characteristics of the general approximation function of the wavelet decomposition, which is the series obtained after the translation and expansion of the data. Because of the translation and expansion, two new parameters–translation factor and expansion factor are generated, and these two parameters give wavelet NN more freedom, so it's easier to approach functions. At the same time, with faster learning ability, the wavelet NN has a stronger data identification ability, and with a relatively simpler network structure, the fault tolerance rate of wavelet NN is higher. Compared with the BPNN algorithm, wavelet NN has better practicability and operability. Structurally, the wavelet NN is a feedforward network, and the process of data processing is completed based on wavelet analysis. Specifically, the data are connected to the NN after the wavelet function changes, and the signal expression is mainly formed by the superposition of wavelet functions. When the signal is classified, the feature space is composed of the wavelet vector space, and the inner product of the wavelet unit and the signal vector is assigned as the feature vector. Finally, the feature vector is classified according to the feature space. Since the feature vector contains both the local variation characteristics of the wavelet function and the self-learning function of the NN, the function approximation of the wavelet NN is very strong.

College students' entrepreneurial psychology is complex and contains many objective and human factors, which have no linear relationship, serious uncertainty, and strong volatility, and these factors will change with time. Although there is domestic and international research on college students' entrepreneurial psychology, the results are very vague. There are no sufficient and conclusive data results to prove the complex relationship between various factors, and there is no specific functional relationship or law between the overall psychological state and various factors, so the study of college students' entrepreneurial psychological effect cannot be solved solely through conventional QS and psychological measurement evaluation reports. The non-linear complex change problem is easier for the AI NN mathematical modeling, and the advantages of wavelet NN, such as super learning ability and good time-frequency localization analysis, can well-predict and evaluate the entrepreneurial psychological effect of college students.

### Experimental Design

The neural network is a mathematical model, which is similar to the human nervous system. It uses a large number of neurons with non-linear mapping ability in the process of processing information and is organized in the network according to the form of a hierarchical structure, so the neural network is a parallel computing system, which is highly interconnected by many simple neurons. The traditional artificial intelligence method is based on symbolic reasoning. Compared with the traditional artificial intelligence method, the neural network has the following characteristics: (1) It has strong nonlinear processing ability, and the theory and time show that the multi-layer neural network model can be highly approximated to any continuous non-linear function; (2) It has high self-adaptation and self-organization ability, and it can continuously absorb information from the external environment, constantly change and organize itself. The adaptive and self-organizing capabilities of neural networks are very useful for subsequent fault diagnosis and prediction. After the neural network completes the learning of the sample data, it can perform abstract analysis and generalization of the training sample data. At this time, the original neural network already contains the summary of the information characteristics of all test sample data. (3) Neural networks are very good at reasoning, analogy, and association. They have powerful classification and screening capabilities, feature expression capabilities, and pattern discrimination capabilities, which enhance the speed and overall nature of information processing.

**Figure 1:**
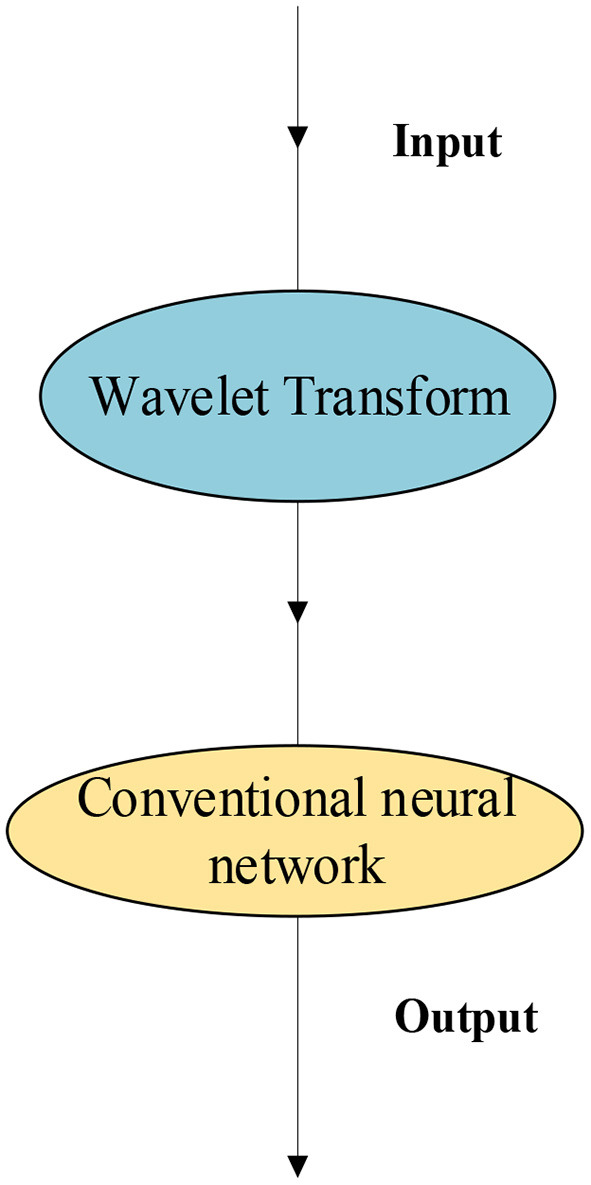
Combination of wavelet transform and NN.

The wavelet NN is formed through the combination of wavelet transform with the NN, in which the wavelet function replaces the excitation function of the hidden layer node of the conventional NN. The weight and threshold of the hidden layer from the corresponding input layer to the hidden layer are replaced by the scale and translation parameters of the wavelet function, respectively. After wavelet transform, the signal is input to the conventional NN for classification and approximation. Here, the samples are selected from last year's entrepreneurial situation of law graduates of university A, and a prediction is made for the entrepreneurial situation of law students. The combination of the wavelet transform and NN is shown in Figure 1.

**Figure 2:**
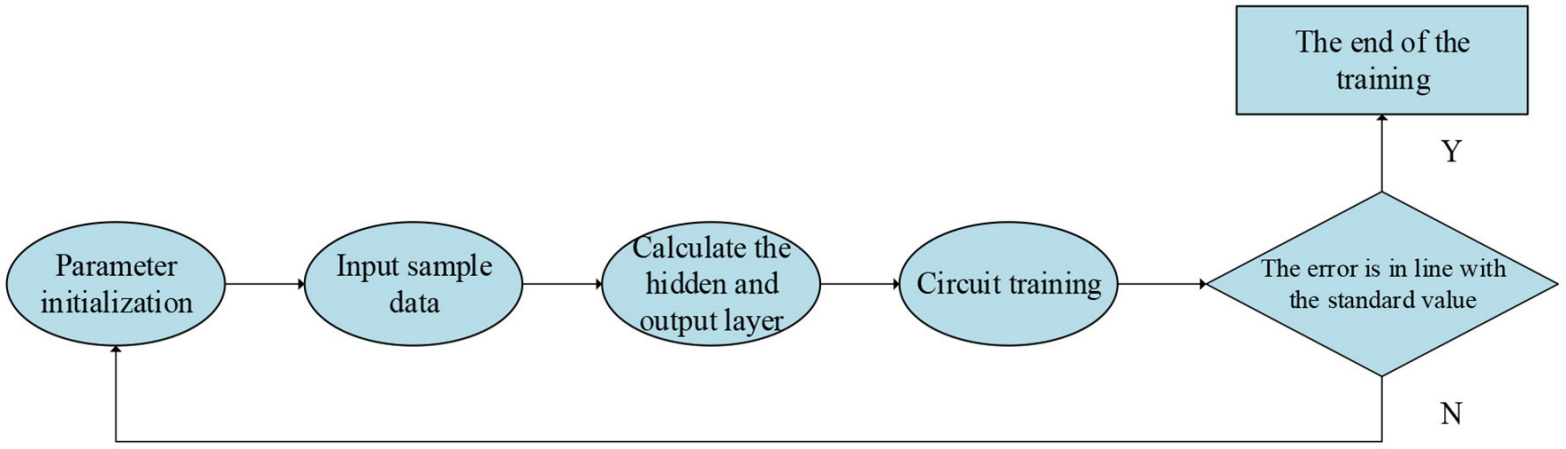
Learning and training process of wavelet transform.

The specific steps of wavelet NN operation are as follows. (1) Network parameter initialization: the parameter of the wavelet NN are randomly generated in the interval (0,1), the network learning rate is set as η = 0.02, and the momentum factor is set as α = 0.935. (2) Then, 20 groups of entrepreneurial psychological samples are input for learning sample and output values, and the top 10 people are the last graduate students and are numbered 1–10, while the last 10 people are new graduates and are numbered 11–20. (3) The hidden layer and output layer nodes are calculated according to the algorithm. (4) Circle training is started and doesn't stop until the total training error meets the given standard or the number of training steps is >10,000 steps. (5) If the total training error satisfies the given standard 0.001, the training parameters are saved, and the training ends. If the total training error doesn't satisfy the given standard, the process returns to step (1). For a better evaluation, the four dimensions of entrepreneurial psychology are trained and analyzed, including entrepreneurial awareness, entrepreneurial volition, entrepreneurial ability, and entrepreneurial personality, as well as the overall evaluation of entrepreneurial psychology. In entrepreneurial awareness training, only the sample data output of entrepreneurial awareness is 1, and the sample data output of the other three dimensions is 0. Similarly, in entrepreneurial volition training, only the sample data output of entrepreneurial volition is 1, and the other three dimensions are 0. In this way, the four dimensions are trained alternately, and finally, the experience and professional knowledge are stored in the NN. The specific process is shown in Figure 2.

### Survey Design

Combining the analysis of the psychological characteristics of contemporary college students' entrepreneurship, and through QS and other methods, it is possible to understand the usual psychological performance and entrepreneurial psychology of law college students. Based on the above theory, it is determined that the constituent elements of college students' entrepreneurial psychological quality are four dimensions: entrepreneurial awareness, entrepreneurial will, entrepreneurial ability, and entrepreneurial personality. In scientific research, in order to collect as many variables as possible that reflect the research object and have a more comprehensive and complete grasp and understanding of the problem, it is necessary to collect a large sample of multiple variables to provide information for scientific research. Factor analysis aims to find a few comprehensive indicators from many related indicators to reflect the main information contained in the original indicators. It uses a few factors to describe the relationship between many indicators or factors, which is a statistical method that reflects most of the information in the original data.

The main content of the QS includes basic personal ability information (gender, age, major, educational background, etc.), a four-dimensional scale of entrepreneurial psychology of college student entrepreneurs, a total of 49 questions, the “Entrepreneurship Motivation Structure Measurement Scale” compiled by Kuratko et al. (1997) and Robichaud (2001) Entrepreneurship Motivation Scale. The item uses a statement method, including positive statements and negative statements, using a 5-point scoring method. It should be noted that before performing the following statistical analysis, first, convert the scores of the reverse question item according to the idea to make it consistent with the direction of other items in the dimension so that it's more convenient when calculating the average of the dimensions later. The non-named scale is adopted, the test survey is conducted by one person, the unified instruction is used, and the QS is divided into 50 points. After the QS is collected, the options of each question in each QS are coded as numeric characters, and the specific code is in the basic information part, the “male” code of the first question is “1,” and the “female” code is “2.” The choice code of each item is 1, 2, 3, and 4 in order. The social statistics software SPSSI17.0 is used to analyze the recovered data. The analysis methods used include factor analysis, descriptive statistics, and correlation analysis.

#### Factor Analysis of Reliability and Validity of QS

Concept of reliability: if T represents the real score, B denotes the deviation score (namely, systematic error), and E stands for the measurement error (namely, random error), and X is the actual score measured in the QS (namely, the observation score), then:


(3) $$\begin{array}{l} X=T+B+E \end{array}$$

A real score is an abstract concept. In other words, the actual-measured score X and the real score T of a potential variable cannot be completely consistent. There will always be errors. Random error cannot be avoided, and the systematic error should be avoided or reduced as much as possible. Since it is difficult to decompose the systematic error in the actual QS, the decomposition equations in some literature references include the systematic error in the real value, that is, Equation (3) is simplified as Equation (4):


(4) $$\begin{array}{l} X=T+E \end{array}$$

Generally, the expected value of the measurement error is assumed to be zero, which is independent of the real score. Under this assumption, it can be proved that the real score is equal to the overall mean of the measured score:


(5) $$\begin{array}{l} E\left(X\right)=E\left(T\right) \end{array}$$

(6) $$\begin{array}{l} \sigma_{X}^{2}=\sigma_{T}^{2}+\sigma_{E}^{2} \end{array}$$

Commonly, reliability is defined as the proportion of the variance of the real score in the total variance, and the mathematical expression reads:


(7) $$\begin{array}{l} y_{XX}=\frac{\sigma_{T}^{2}}{\sigma_{X}^{2}}\left(1-\frac{\sigma_{E}^{2}}{\sigma_{X}^{2}}\right) \end{array}$$

Or, it can be defined as:


(8) $$\begin{array}{l} y_{XX}=\sqrt{\frac{\sigma_{T}^{2}}{\sigma_{X}^{2}}} \end{array}$$

Equation (7) expresses the reliability as a form of the correlation coefficient: the correlation between the real score and the actual-measured score; Equation (8) expresses the reliability as a variation ratio, which is the square of the correlation coefficient, or as the decisive coefficient between the real score and the actual-measured score. It is very similar to the determination coefficient introduced in the regression ANOVA (Analysis of Variance).

#### Reliability Coefficient

Reliability analysis software can study the internal reliability of the scale. Firstly, the analytical software makes a basic description of each evaluation item, calculates the simple correlation coefficient of each item and items other than the excluded single item. The internal reliability is analyzed. Then, various reliability analysis coefficients are used to further study the internal reliability or external reliability. If the reliability coefficient is used to represent the reliability, the greater the reliability coefficient is, the greater the reliability of the measurement is. For the ability and achievement test, the measured reliability coefficient should be >0.9 to be effective. A well-designed QS can reach a 0.95 reliability coefficient. For personality tests, such as personality, interest, and values, it is more appropriate that the measured reliability coefficient is >0.8 and <0.85.

The validity, namely effectiveness, refers to the accurately measurable degree of a specific item using measuring tools or means. In other words, it is the degree to which the measured results reflect the investigated content. The higher the validity is, the higher the degree to which the measured results tend to be consistent with the content to be investigated; on the contrary, the lower the validity is, the lower the degree of consistency between the measurement results and the content to be investigated. Validity can be divided into three types: criterion validity, content validity, and structure validity. Scientific measuring tools must satisfy such conditions as high efficiency. As a social measurement tool, QS or scale has high requirements for validity. To measure validity, one should analyze the purpose and scope of the investigation and clarify and check whether the content is consistent with the purpose of the investigation. Meanwhile, the content should also be analyzed, as well as its nature and characteristics, and the results are verified to see whether they can reflect the characteristics of the measurement.

**Figure 3:**
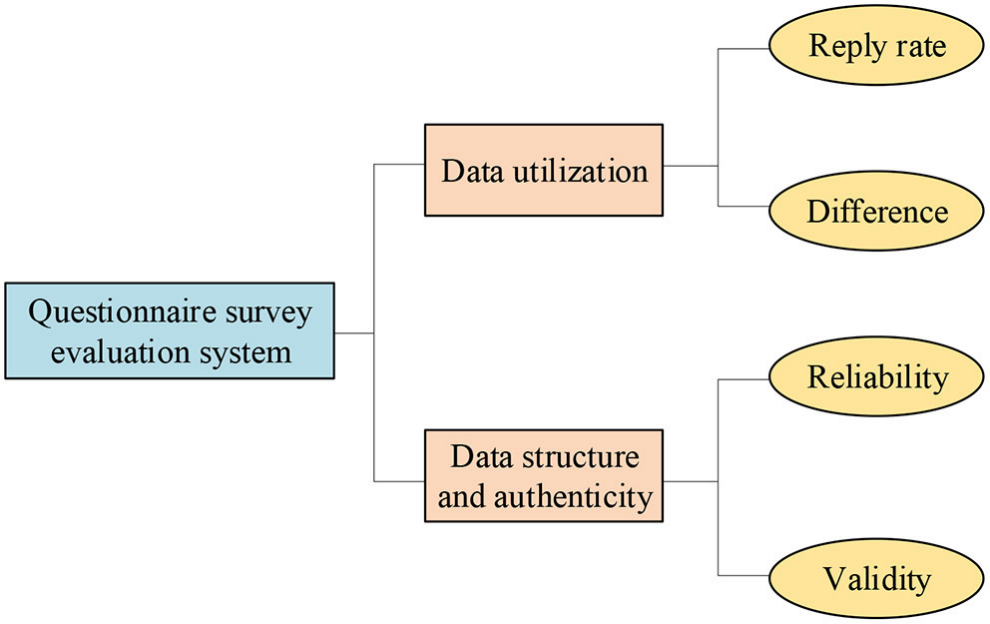
Structure diagram of QS evaluation system.

The evaluation results should be reliable and accurate. Therefore, the indicators should be selected to be as independent as possible. The indicator system can be used for target evaluation, during which information reuse should be avoided, the indicator should be mutually independent, and the indicator system should be scientific and reasonable. The QS evaluation indicator system is shown in Figure 3.

## Experimental Analysis of Wavelet NN

### QS Reliability and Validity Test

**Figure 4:**
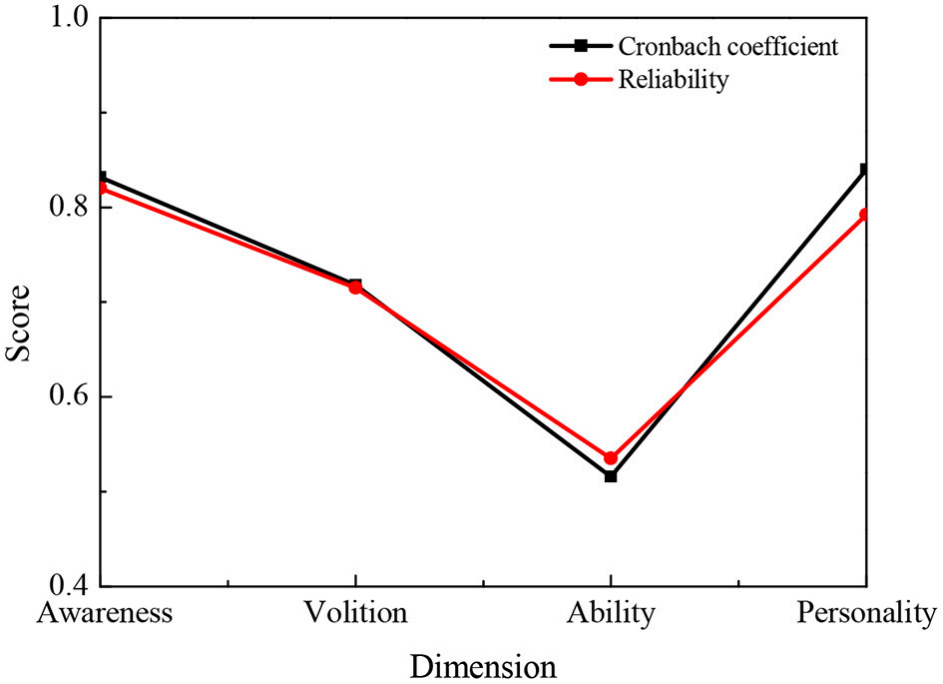
Reliability analysis of college students' entrepreneurial psychology QS.

Through the analysis of the QS data, the internal consistency coefficient (Cronbach's α coefficient) and the results of reliability are shown in Figure 4.

Figure 4 implies that the overall internal consistency coefficient of college students' entrepreneurial psychology QS is high, which is 0.82; the split-half reliability is 0.795, but from a single dimension, the internal consistency coefficient of entrepreneurial awareness and entrepreneurial personality is high, while the coefficient of entrepreneurial ability is low, indicating that the designed questions for entrepreneurial awareness and entrepreneurial personality have good reliability.

### The Prediction Results of Entrepreneurial Psychology

**Figure 5:**
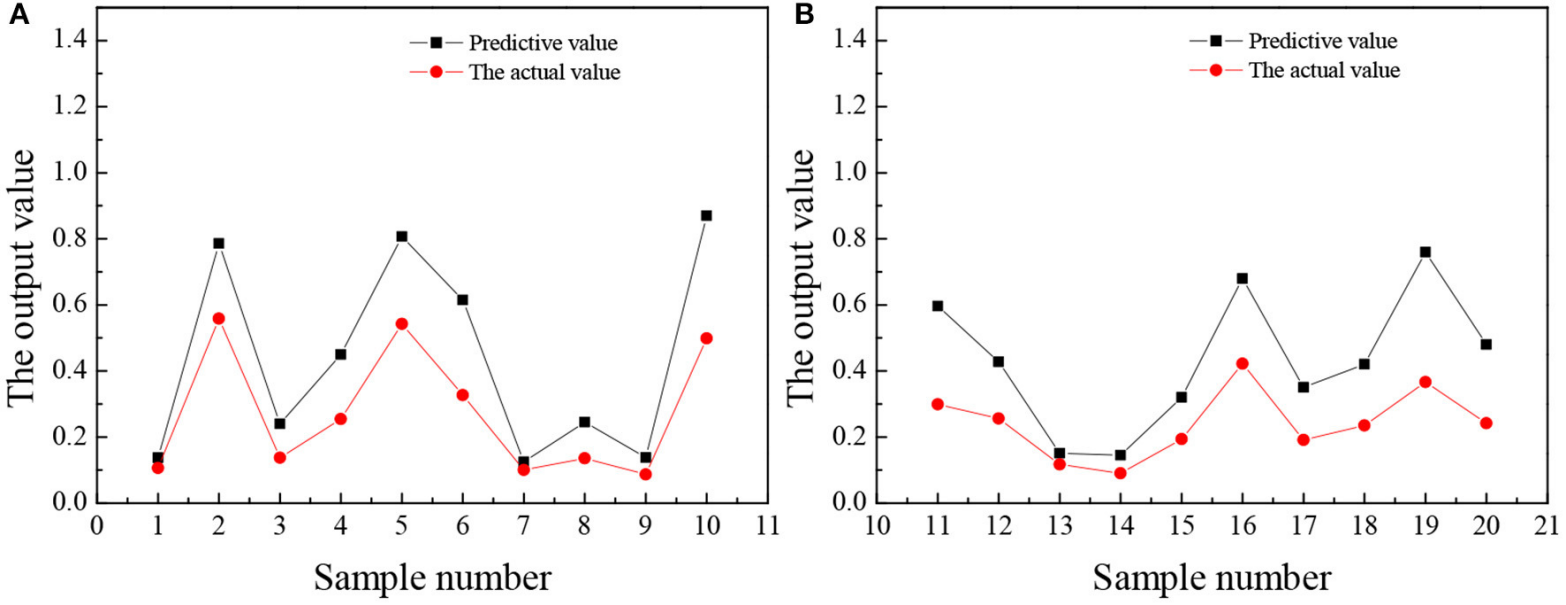
The comparison between the predicted value and the actual value of entrepreneurial awareness **(A)** No. 1–10 **(B)** No. 11–20.

**Figure 6:**
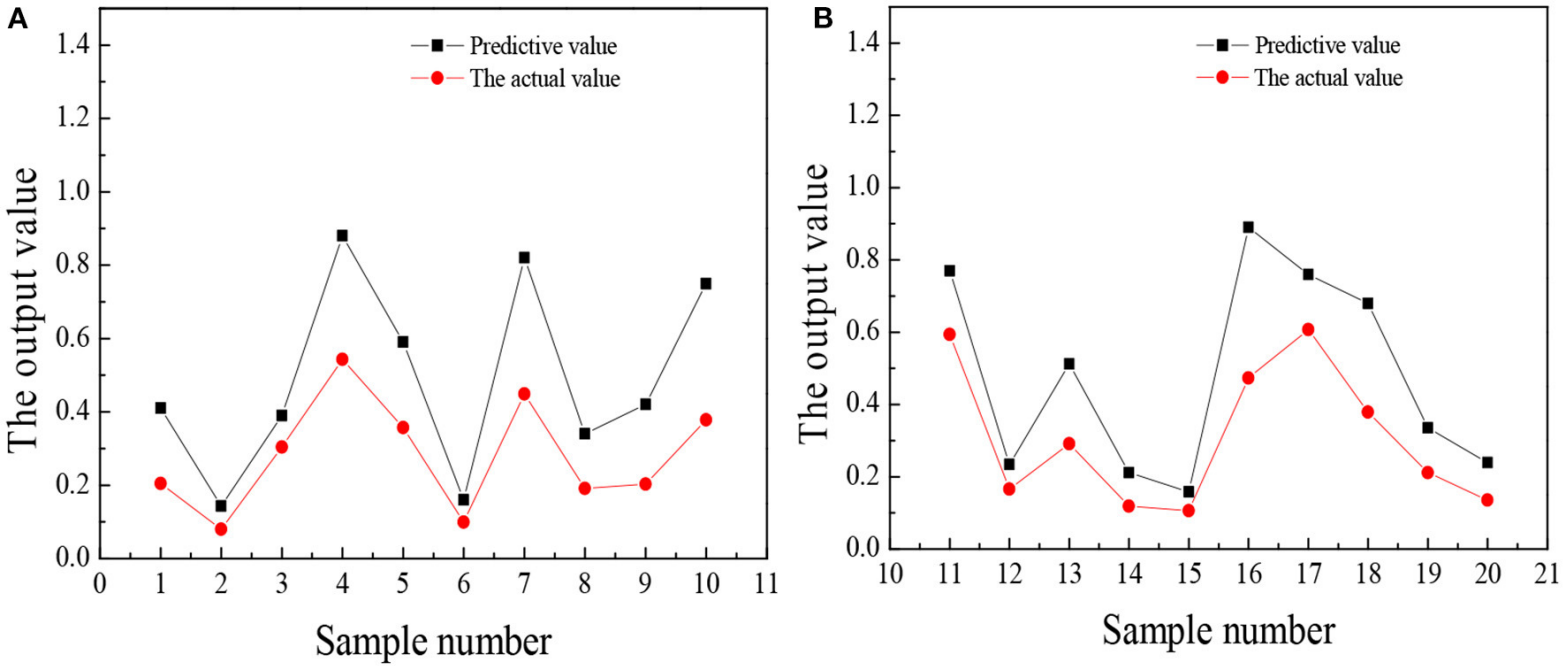
The comparison between the predicted value and the actual value of entrepreneurial volition **(A)** No. 1–10 **(B)** No. 11–20.

**Figure 7:**
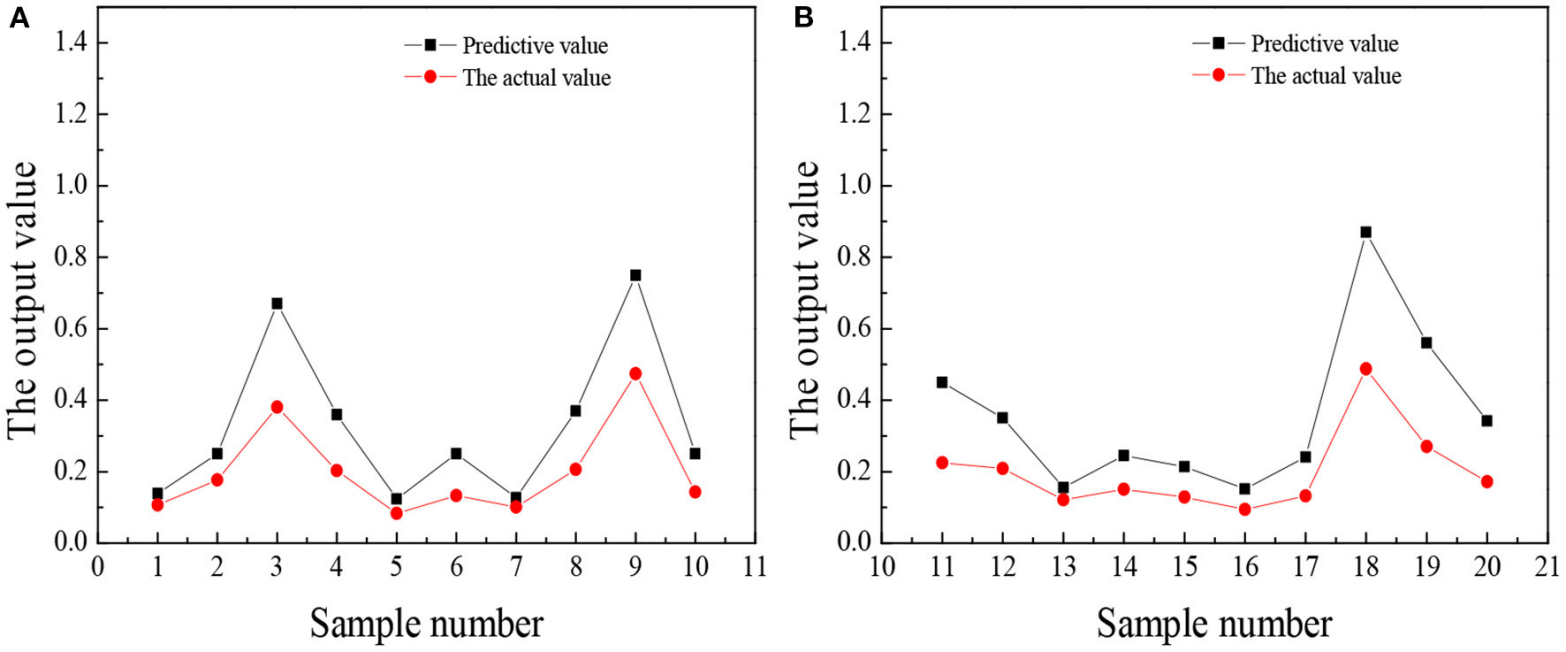
The comparison between the predicted value and the actual value of entrepreneurial ability **(A)** No. 1–10 **(B)** No. 11–20.

**Figure 8:**
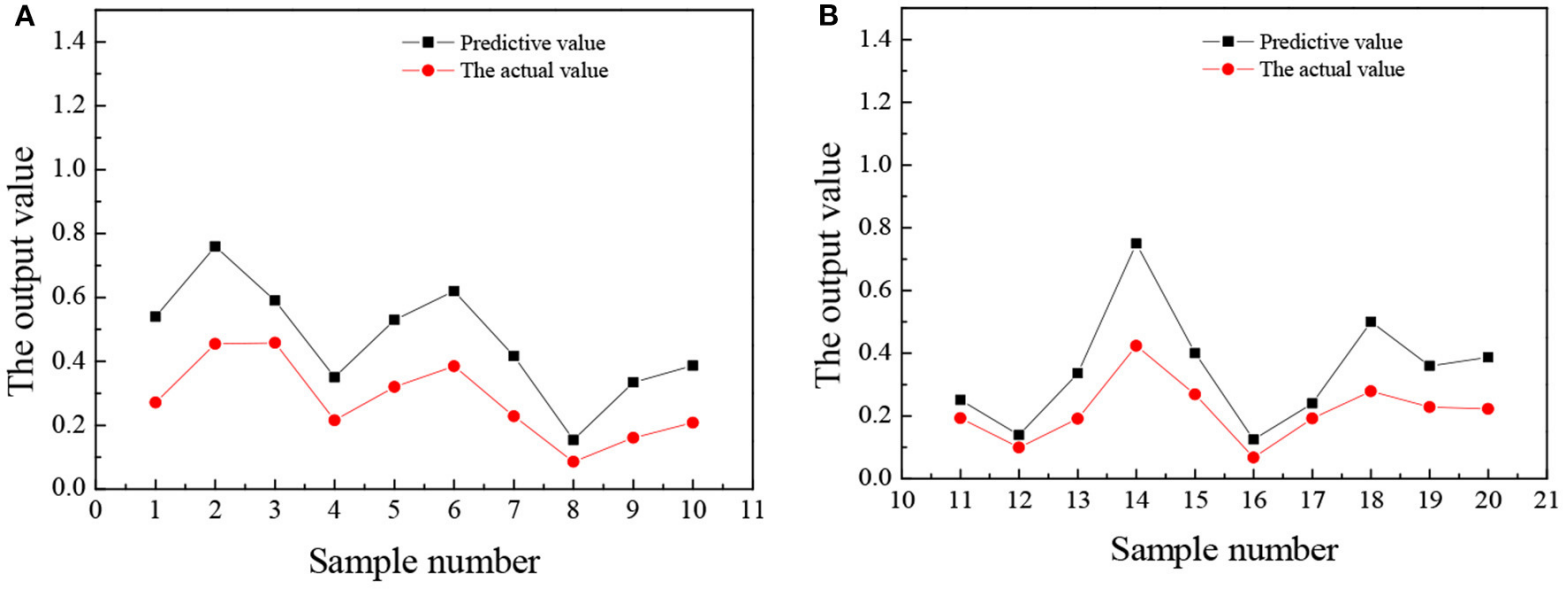
The comparison between the predicted value and the actual value of entrepreneurial personality **(A)** No. 1–10 **(B)** No. 11–20.

The trained wavelet NN predicts the entrepreneurial awareness, entrepreneurial volition, entrepreneurial ability, and entrepreneurial personality of 20 law students in university A, and the results are shown in Figures 5–**8**.

Figure 6 suggests that the predicted value of college students' entrepreneurial awareness is higher than the actual value. Most college students choose to start a business without in-depth understanding and serious thinking. Instead, they choose to start a business on a whim, which will lead to a serious lack of follow-up motivation. Additionally, many college students are not firm in their will to start a business and choose to retreat when they encounter problems. Such a business often ends in a failure.

Figure 7 illustrates that the predicted value of college students' entrepreneurial will is about 0.2 points higher than the actual value. The similarity between the overall prediction and the actual trend is very high. The entrepreneurial will of students is very different, so the accuracy of the predicted value is also very high. The overall score of entrepreneurial will is low. The reason may be that if they choose to start a business, they will face many difficulties and challenges. Most college students cannot independently solve the problems encountered in the process of starting a business through their own efforts, and finally, give up. This shows that most college students lack perseverance and willpower and do not have good entrepreneurial psychological qualities.

Figure 8 reveals that the predicted value of college students' entrepreneurial personality is not very different from the actual value. Overall, the predicted value is about 0.3 points higher than the actual value, but from the score of entrepreneurial personality, the accuracy of the predicted value is very high. Most college students will choose a stable route on the road of entrepreneurship, such as choosing to enter the service industry with a low threshold. Few people can think of seeking breakthroughs or trying new ideas and lack an adventurous and entrepreneurial spirit. In the long run, this will restrict the sustainable development of college students entrepreneurship. Figures 5–8 suggest that the students numbered 2, 5, 10, 16, and 19 have strong entrepreneurial awareness; students numbered 4, 7, 10, 11, 13, and 16 have a stronger entrepreneurial volition; students numbered 3, 9, and 18 are better entrepreneurial ability, while the entrepreneurial personality of students numbered 2, 14, and 18 are more outstanding. On the whole, the predicted value of students' entrepreneurial psychology is higher than the actual value, but the deviation is not large, which is closer to the actual value and the overall prediction is consistent with the actual situation. The results prove that the prediction analysis of AI technology on college students' entrepreneurial psychology is feasible.

### Single-Factor Analysis of Entrepreneurial Psychology

**Figure 9:**
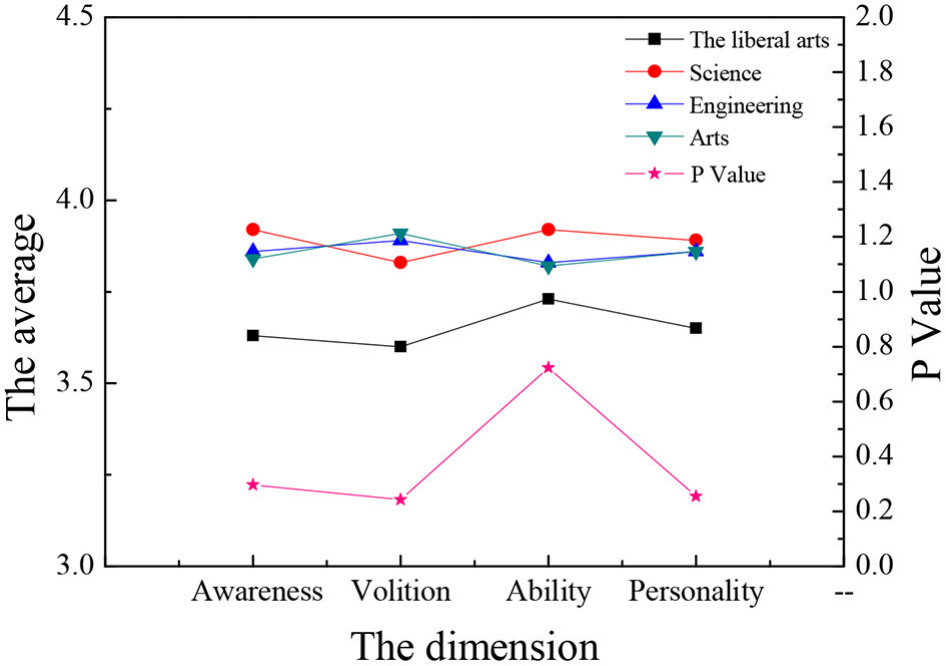
The average value of each dimension of entrepreneurial psychology of students of different majors.

**Figure 10:**
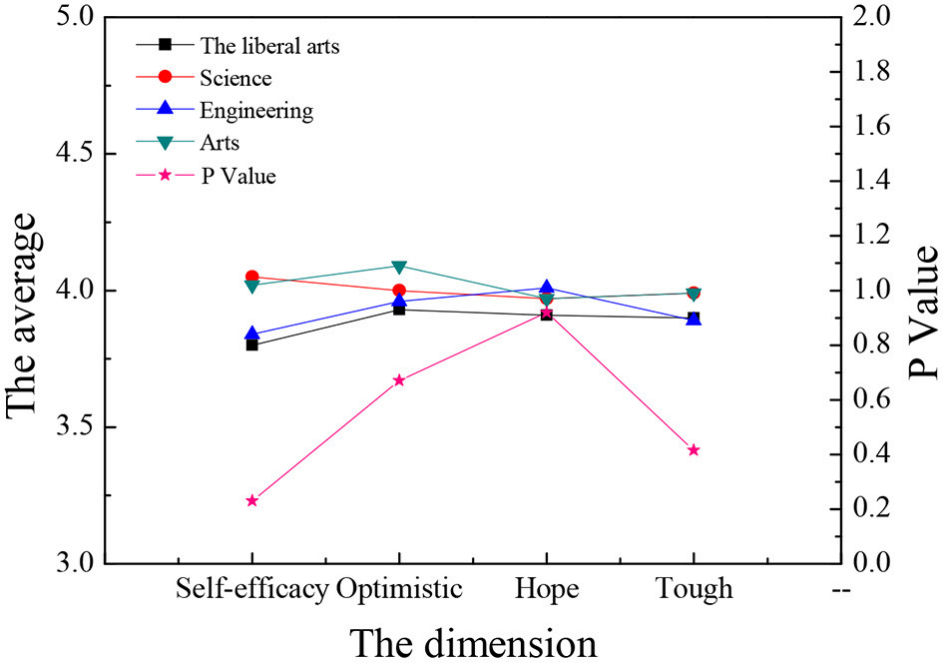
Comparison of entrepreneurial psychological capital of different majors.

**Figure 11:**
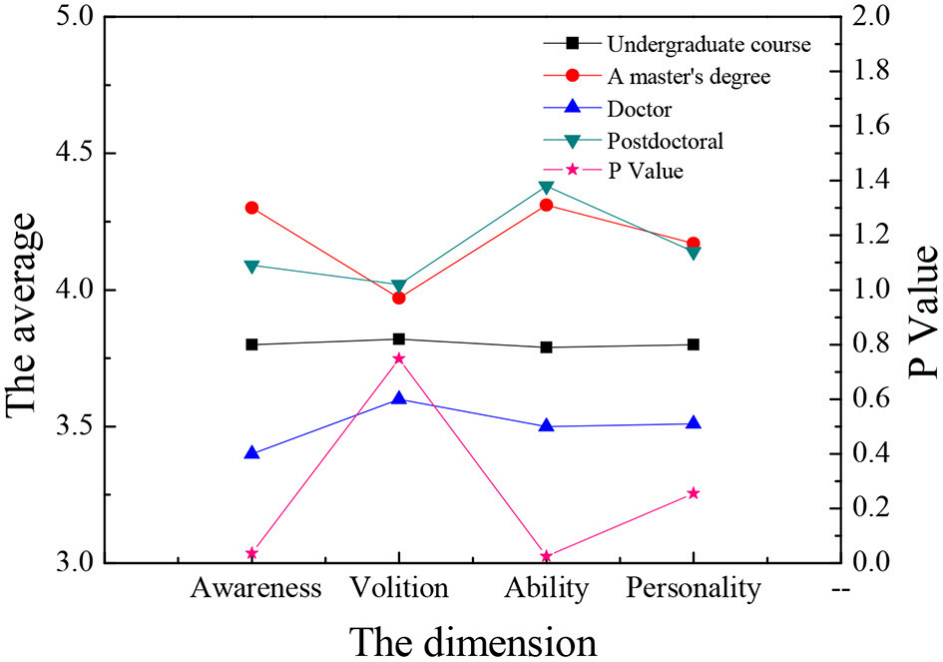
Comparative study on entrepreneurial psychology of law students with different educational backgrounds.

**Figure 12:**
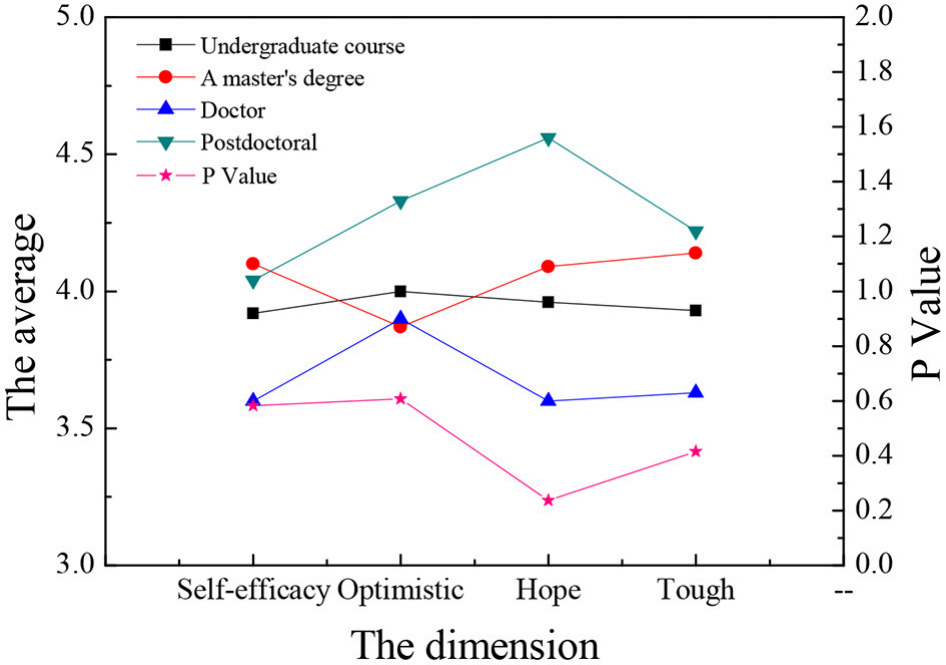
Comparison of psychological capital of law students with different educational background.

**Figure 13:**
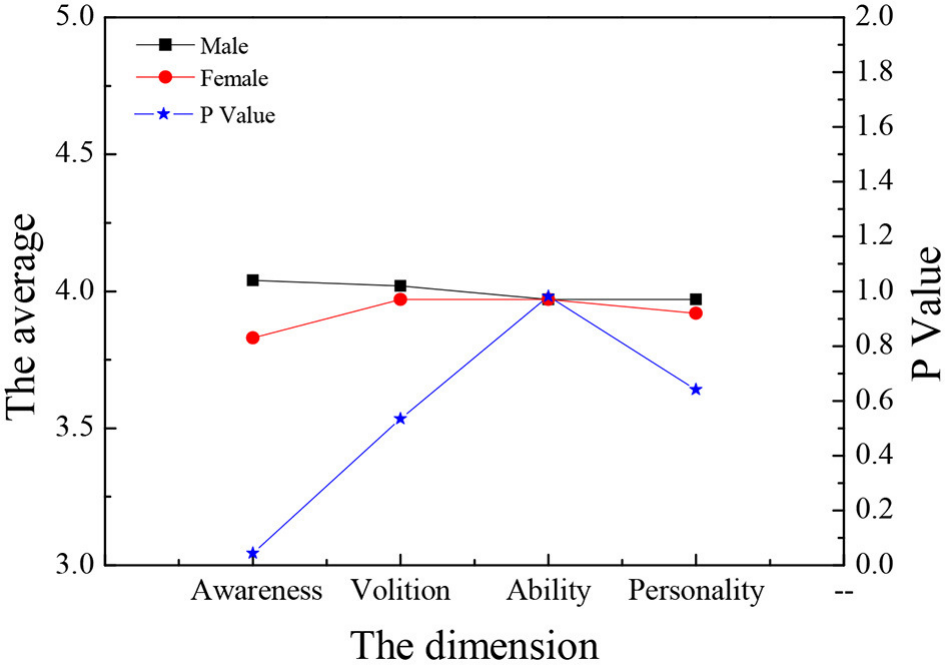
Comparison of entrepreneurial psychology of law students of different genders.

**Figure 14:**
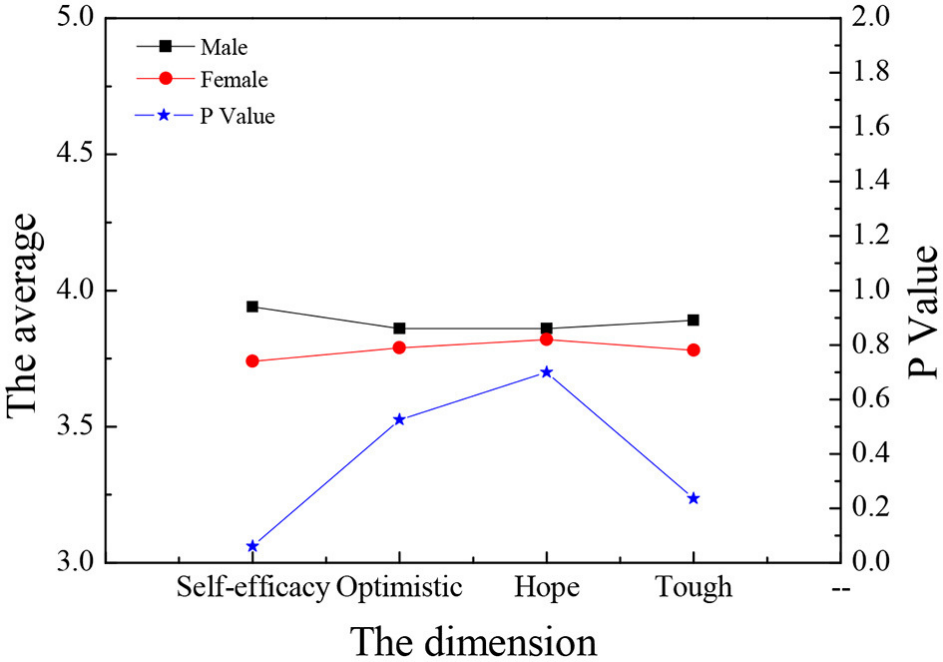
Comparison of entrepreneurial psychological capital of law students of different genders.

The sample data are analyzed through single-factor ANOVA (Analysis of Variance), and the ANOVA is carried out according to the three dimensions: gender, major, and educational background. Meanwhile, the four dimensions of entrepreneurial psychological capital are analyzed and compared. The results are shown in Figures 9–**14**.

Figures 9, 10 reveal that: although students of different disciplines have different scores on the four dimensions of entrepreneurial psychology, in terms of entrepreneurial intention, science students score the highest and liberal arts students the lowest; in terms of the entrepreneurial will, art students score the highest and liberal arts students the lowest; in entrepreneurial ability and entrepreneurial will, science students score the highest and the liberal arts students the lowest; but these differences are not obvious (*P* > 0.05), which shows that the major of college students is not the main factor affecting their entrepreneurial psychology. There is no significant difference in the four aspects of entrepreneurial psychological capital among different majors (*P* > 0.05).

Figures 11, 12 demonstrate that there are significant differences in entrepreneurial psychology among law students with different educational backgrounds (*P* < 0.05), among which students with master's degrees have the strongest entrepreneurial volition, while the doctoral students have the lowest entrepreneurial volition. The results imply that students with master's degrees have greater confidence in entrepreneurship and have greater advantages in preparing entrepreneurial resources when the knowledge reserve is sufficient. In the other three dimensions, there is no significant difference between different educational backgrounds (*P* > 0.05). Students of all grades have similarities in entrepreneurial ability, entrepreneurial volition, and entrepreneurial personality. In terms of psychological capital, there is no significant difference between self-efficacy, optimism, hope, and toughness (*P* > 0.05), which indicates that educational background is not the main influencing factor of college students' entrepreneurial psychological capital, and there is no great difference in self-energy efficiency, optimism, hope, and resilience.

Figures 13, 14 illustrate that there is no significant difference between the males and females in the four aspects of entrepreneurship psychology (*P* > 0.05). In terms of entrepreneurial psychological capital, the self-efficacy of males is higher than that of females, and the difference is significant (*P* < 0.05). The other three dimensions are not so different. As a whole, gender has no great influence on entrepreneurship psychology.

## Conclusions

Due to the lack of education on the psychological quality of entrepreneurship, college students are prone to psychological fluctuations, resulting in college students' lack of self-confidence in entrepreneurship, and some even choose to give up halfway. Therefore, there is a need to strengthen the cultivation of college students' entrepreneurial psychological quality and cultivate college students with positive entrepreneurial psychological quality. Here, based on AI technology, the wavelet transform and NN are combined for circle training to analyze and predict the four dimensions of college students' entrepreneurial psychology and verify the prediction results. The results show that the predicted value of wavelet NN is consistent with the actual value, and the application of AI in College Students' entrepreneurial psychology is feasible. Overall, the difference between the predicted value and the actual value is within 0.3 points, which is relatively stable. The single-factor analysis of college students' entrepreneurial psychology reveals that different educational background has a greater impact on the entrepreneurial volition of law students, while gender difference has a significant impact on student's self-efficacy. The scores difference between males and females in the four aspects of entrepreneurial psychological factors is not very obvious. In terms of entrepreneurial psychological capital, males' self-efficacy is significantly higher than females (*P* < 0.05). Meanwhile, there are still some shortcomings: the QS “Investigation on Entrepreneurship Psychological Quality of College Students” is compiled on the basis of the existing domestic and foreign QS on the entrepreneurial psychological quality of entrepreneurs. The reliability and validity of the QS still have certain limitations. In addition, the questions involved in the five scales of this study are too small, which cannot cover all the content of college students' entrepreneurial psychological quality. Subsequent research can further enrich the content of the QS. With the continuous progress of AI technology, NN algorithms will continue to improve, thus predicting data more accurately to help higher education institutions further understand the entrepreneurial psychology of students. With changes in environment and time, entrepreneurial psychological quality will show changes. Follow-up research can longitudinally track the entrepreneurial psychological quality and influencing factors of college students at different times and in different environments, thus enriching and perfecting the content of this research.

## Data Availability Statement

The raw data supporting the conclusions of this article will be made available by the authors, without undue reservation.

## Ethics Statement

The studies involving human participants were reviewed and approved by Shandong Normal University Ethics Committee. The patients/participants provided their written informed consent to participate in this study. Written informed consent was obtained from the individual(s) for the publication of any potentially identifiable images or data included in this article.

## Author Contributions

CX: conceptualization, methodology, and writing–original draft. ZZ: software, supervision, data curation, project administration, and resources. Both authors contributed to the article and approved the submitted version.

## Funding

This work was funded by Shandong Normal University Young Teachers Research Project (Humanities and Social Sciences).

## Conflict of Interest

The authors declare that the research was conducted in the absence of any commercial or financial relationships that could be construed as a potential conflict of interest.

## Publisher's Note

All claims expressed in this article are solely those of the authors and do not necessarily represent those of their affiliated organizations, or those of the publisher, the editors and the reviewers. Any product that may be evaluated in this article, or claim that may be made by its manufacturer, is not guaranteed or endorsed by the publisher.

## References

[B1] Carvalho N. A. T. Carvalho J. G. Souza D. Madureira E. H. Baruselli P. S. (2021). Lack of effect of melatonin on ovarian function and response to estrous synchronization and fixed-time AI during the nonbreeding season in lactating dairy buffalo (*Bubalus bubalis*). Anim. Reprod. Sci. 231:103. 10.1016/j.anireprosci.2021.106796 34174498

[B2] Chen W. Q. Zhang R. Liu H. Xie X. Yan L. (2021). Corrigendum to “A novel method for solar panel temperature determination based on a wavelet neural network and Hammerstein-Wiener model”. Adv. Space Res. 67:177. 10.1016/j.asr.2021.03.009

[B3] Chen Z. Yu X. (2020). Adoption of human personality development theory combined with deep neural network in entrepreneurship education of college students. Front. Psychol. 11:242. 10.3389/fpsyg.2020.01346 32733313 PMC7361131

[B4] Daniella D. T. Cecile N. Chris S. (2021). Entrepreneurial education and individual entrepreneurial orientation: an experts' perspective. An empirical Delphi study. EUREKA Soc. Human. 12, 2760–2770. 10.17762/turcomat.v12i3.1305

[B5] Du W. Zhang Q. Chen Y. Ye Z. (2021). An urban short-term traffic flow prediction model based on wavelet neural network with the improved whale optimization algorithm. Sustain. Cities Soc. 69:102858. 10.1016/j.scs.2021.102858

[B6] Fan Z. Ji P.-p. Zhang J. Segets D. Chen D.-R. Chen S.-C. (2021). Wavelet neural network modeling for the retention efficiency of sub-15 nm nanoparticles in ultrafiltration under small particle to pore diameter ratio. JMS. 635:431. 10.1016/j.memsci.2021.119503

[B7] Guo L. Yang Z. Zhang Q. (2021). Identification of autonomous nonlinear dynamical system based on discrete-time multiscale wavelet neural network. Neural Comput. Appl. 31:38. 10.1007/s00521-021-06142-z

[B8] Guo S. S. Guo S. (2020). Analysis of the status quo of employment and entrepreneurship of college students in the new era based on big data analysis and discussion. JPCS 1648:378. 10.1088/1742-6596/1648/3/032038

[B9] Hgg G. A. (2020). Guiding the student entrepreneur – considering the emergent adult within the pedagogy-andragogy continuum in entrepreneurship education. Educ. Train 62, 22–24. 10.1108/ET-03-2020-0069

[B10] Huang Y. An L. Wang J. Chen Y. Wang P. (2021). The role of entrepreneurship policy in college students' entrepreneurial intention: the intermediary role of entrepreneurial practice and entrepreneurial spirit. Front. Psychol. 12:585698. 10.3389/fpsyg.2021.585698 33776829 PMC7988310

[B11] Huang Y. Li C. (2021). Accurate heating, ventilation, and air conditioning system load prediction for residential buildings using improved ant colony optimization and wavelet neural network. J. Build. Eng. 35:103. 10.1016/j.jobe.2020.101972

[B12] Huang Z. Liu G. Wagner N. (2021). Prediction model of college students entrepreneurship ability based on artificial intelligence and fuzzy logic model. J. Intell. Fuzzy Syst. 40, 2541–2552. 10.3233/JIFS-189247

[B13] Ilyas H. Raja M. A. Z. Ahmad I. Shoaib M. (2021). A novel design of Gaussian wavelet neural networks for nonlinear Falkner-Skan systems in fluid dynamics. Chinese J. Phys. 72:94. 10.1016/j.cjph.2021.05.012

[B14] Jiang H. Zhi W. (2021). Research on the application of the red boat spirit in the innovation and entrepreneurship of college students in higher vocational and technical colleges. J. Simul. 9:230. 10.47939/es.v2i5.01

[B15] Jiang Y. Lan G. Zhang Z. (2021). Ship engine detection based on wavelet neural network and FPGA image scanning. Alex Eng. J. 60:101. 10.1016/j.aej.2021.02.028

[B16] Kuratko D. F. Hornsby J. S. Naffziger D. W. (1997). An examination of owner's goals in sustaining entrepreneurship. J. Small Business Manage. 35. Available online at: https://www.proquest.com/openview/a6cf211c0fcb53e8a16b9732d4ed1665/1?pq-origsite=gscholar&cbl=49244

[B17] Li H. Wang J. Zhang Y. (2020). The impact of self-efficacy analysis-based psychological theory and literary ethics on Chinese American Entrepreneurship Education. Front. Psychol. 11:194. 10.3389/fpsyg.2020.01870 32849097 PMC7417519

[B18] Li M. Wang T. Wu Y. (2021). Impact of innovation and entrepreneurship education in a university under personality psychology education concept on talent training and cultural diversity of new entrepreneurs. Front. Psychol. 69:32. 10.3389/fpsyg.2021.696987 34393928 PMC8358120

[B19] Mu K. Shi Q. Ma Y. Tan J. (2020). Exploration of entrepreneurship education by linear regression and psychological factor analysis. Front. Psychol. 11:2045. 10.3389/fpsyg.2020.02045 32903411 PMC7434862

[B20] Nikpanah M. Xu Z. Jin D. (2021). A deep-learning based artificial intelligence (AI) approach for differentiation of clear cell renal cell carcinoma from oncocytoma on multi-phasic MRI. Clin. Imaging 77:253. 10.1016/j.clinimag.2021.06.016 34171743 PMC9990181

[B21] Ouyang K. Hou Y. Zhou S. Zhang Y. (2021). Adaptive multi-scale wavelet neural network for time series classification. Information 12:42. 10.3390/info12060252

[B22] Ren Z. Liu T. Liu G. (2021). Classification and discrimination of real and fake blood based on phooacoustic spectroscopy combined with particle swarm optimized wavelet neural networks. Photoacoustics 23:42. 10.1016/j.pacs.2021.100278 34141580 PMC8188063

[B23] Robichaud D. (2001). La création d'entreprises par les immigrants: le cas des Québécois d'origine portugaise de la région métropolitaine de recensement de Montréal. Les Nouveaux Cahiers Du Conseil Constitutionnel. 57(1):115–120. 10.3917/nccc.030.0077

[B24] Sabir Z. Nisar K. Raja M. A. Ibrahim A. Rawat D. B. Asri Bin A.. (2021). Design of Morlet wavelet neural network for solving the higher order singular nonlinear differential equations. Alex Eng. J. 60, 5935–5947. 10.1016/j.aej.2021.04.001

[B25] Sergent K. Lee D. Stajkovic A. D. Greenwald J. M. Younger S. Raffiee J. (2020). The mitigating role of trait core confidence on psychological distress in entrepreneurship. Appl. Psychol. 69:146. 10.1111/apps.12267

[B26] Shaheen N. Ahmad N. Munir N. Hussain S. (2020). Psychology of learning entrepreneurship skills: nurturing learning styles of students. Rawal Med. J. 45, 88–191. 10.31703/grr.2019(iv-iv).14

[B27] Sivakumar S. Gopalai A. A. Lim K. (2021). Joint angle estimation with wavelet neural networks. Sci. Rep. 11, 122–123. 10.1038/s41598-021-89580-y 33986396 PMC8119494

[B28] Sukirno S. Zahran W. S. Tamba R. Suparman S. (2020). Personality traits to develop entrepreneurship skills of college students: a case study of student entrepreneurial program. Tec. Soc. Sci. J. 12:321. 10.24940/theijbm/2020/v8/i12/bm2012-002

[B29] Ting D. (2020). Exploration of innovative course teaching of the theme hotel design—take the college students' entrepreneurship program of environmental design major as an example. Int. J. Sci. 7:320. 10.1109/iciddt52279.2020.00095

[B30] Urooj S. Singh S. P. Malibari A. (2021). Early detection of Alzheimer's disease using polar harmonic transforms and optimized wavelet neural network. Appl. Sci. 11:290. 10.3390/app11041574

[B31] Wachter S. Mittelstadt B. Russell C. (2021). Why fairness cannot be automated: bridging the gap between EU non-discrimination law and AI. Comput. Law Security Rev. 41:64. 10.1016/j.clsr.2021.105567

[B32] Wang X. Chai H. Z. Wang C. Xiao G. Guan X. (2021). Improved wavelet neuranetwork based on change rate to predict satellite clock bias. Surv. Rev. 53:31. 10.1080/00396265.2020.1758999

[B33] Wu F. Mao C. (2020). Business environment and entrepreneurial motivations of urban students. Front. Psychol. 11:1483. 10.3389/fpsyg.2020.01483 32848974 PMC7399197

[B34] Wu Y. Song D. (2019). Gratifications for social media use in entrepreneurship courses: learners' perspective. Front. Psychol. 10:1270. 10.3389/fpsyg.2019.01270 31214081 PMC6555126

[B35] Wu Y. Yuan C. H. (2018). Entrepreneurship education: an experimental study with information and communication technology. Sustainability 10:691. 10.3390/su10030691

[B36] Xi H. Wu X. Chen X. Sha P. (2021). Artificial intelligent based energy scheduling of steel mill gas utilization system towards carbon neutrality. Appl. Energ. 12:295. 10.1016/j.apenergy.2021.117069

[B37] Xu M. Chu X. Fu Y. Wang C. Wu S. (2021). Improving the accuracy of soil organic carbon content prediction based on visible and near-infrared spectroscopy and machine learning. Environ. Earth Sci. 80:339. 10.1007/s12665-021-09582-x

[B38] Yin Y. Yang L. Liu B. (2020). Analysis on entrepreneurship psychology of preschool education students with entrepreneurial intention. Front. Psychol. 11:1559. 10.3389/fpsyg.2020.01559 32733339 PMC7363936

[B39] Yuan C. H. Wang D. M. Chuan W. F. (2020). An empirical comparison of graduate entrepreneurs and graduate employees based on graduate entrepreneurship education and career development. Sustainability 12:231. 10.3390/su122410563

[B40] Zhang B. Xu Q. Han S. Jiao L. (2020). Analysis on influences of college students' psychological capital in entrepreneurial learning engagement. Front. Psychol. 11:361. 10.3389/fpsyg.2020.02029 33013510 PMC7461884

[B41] Zheng W. Qian F. Zhao S. Zhang Y. (2021). M-GWNN: Multi-granularity graph wavelet neural networks for semi-supervised node classification. Neurocomputing 453:42. 10.1016/j.neucom.2020.10.033

